# Antibacterial, antibiofilm, and cytotoxic activities and chemical compositions of Peruvian propolis in an *in vitro* oral biofilm

**DOI:** 10.12688/f1000research.73602.2

**Published:** 2021-12-23

**Authors:** Pablo Alejandro Millones Gómez, Lidia Yileng Tay Chu Jon, Dora Jesús Maurtua Torres, Reyma Evelyn Bacilio Amaranto, Ingrit Elida Collantes Díaz, Carlos Alberto Minchón Medina, Jaeson Santos Calla Choque

**Affiliations:** 1 Facultad de Medicina, Universidad Señor de Sipán, Chiclayo, 14000, Peru; 2 Faculty of Dentistry, Universidad Peruana Cayetano Heredia, Lima, 07001, Peru; 3 Faculty of Science, Universidad Peruana Cayetano Heredia, Lima, 07001, Peru; 4 Facultad de Ingeniería Química y Textil, Universidad Nacional de Ingeniería, Lima, 07001, Peru; 5 Department of Statistics, Faculty of Physical Sciences and Mathematics, Universidad Nacional de Trujillo, Trujillo, 13001, Peru; 6 Department of Pediatrics, School of Medicine, University of California San Diego (UCSD), La Jolla, CA, 92093, USA

**Keywords:** oral biofilm, propolis, antibacterial, antibiofilm, chemical compositions

## Abstract

**Background:** Natural products with antibacterial potential have begun to be tested on biofilm models, bringing us closer to understanding the response generated by the complex microbial ecosystems of the oral cavity. The objective of this study was to evaluate the antibacterial, antibiofilm, and cytotoxic activities and chemical compositions of Peruvian propolis in an *in vitro* biofilm of *Streptococcus gordonii *and *Fusobacterium nucleatum*.

**Methods:** The experimental work involved a consecutive, *in vitro*, longitudinal, and double-blinded study design. Propolis samples were collected from 13 different regions of the Peruvian Andes. The disk diffusion method was used for the antimicrobial susceptibility test. The cytotoxic effect of propolis on human gingival fibroblasts was determined by cell viability method using the MTT (3-[4,5-dimethylthiazol-2-yl]-2,5 diphenyl tetrazolium bromide) assay, and the effect of propolis on the biofilm was evaluated by confocal microscopy and polymerase chain reaction (PCR).

**Results:** The 0.78 mg/mL and 1.563 mg/mL concentrations of the methanolic fraction of the chloroform residue of Oxapampa propolis showed effects on biofilm thickness and the copy numbers of the *srtA* gene of *S. gordonii* and the *radD* gene of *F. nucleatum* at 48 and 120 hours, and chromatography (UV, λ 280 nm) identified rhamnocitrin, isorhamnetin, apigenin, kaempferol, diosmetin, acacetin, glycerol, and chrysoeriol.

**Conclusions: **Of the 13 propolis evaluated, it was found that only the methanolic fraction of Oxapampa propolis showed antibacterial and antibiofilm effects without causing damage to human gingival fibroblasts. Likewise, when evaluating the chemical composition of this fraction, eight flavonoids were identified.

## Introduction

*Fusobacterium nucleatum* ( Choi *et al*., 2020; Geng *et al*., 2018) and *Streptococcus gordonii* ( Geng *et al*., 2018) are some of the most important bacteria in periodontal and peri-implant disease, especially in patients who are smokers ( Lazar *et al*., 2015). The scientific community has made considerable efforts to discover new antimicrobial agents that can disrupt the complex structures of subgingival biofilms. One of the main sources of new molecules with antimicrobial activity is products of natural origin ( Arbia *et al*., 2017; Lee *et al*., 2017; Slobodníková *et al*., 2016). The search for new antimicrobial agents has gained momentum, particularly due to increased rates of antibiotic resistance to periodontal pathogens ( Kouidhi *et al*., 2015; Rams *et al*., 2014; Van Winkelhoff *et al*., 2005).

Socransky *et al*. (1988) found that the subgingival microbiota can be classified into different complexes based on the association of different microbial species with healthy or diseased sites. If left intact, gingival biofilms create conditions that can favour colonization by orange-complex species, namely, *Fusobacterium, Campylobacter*, *Prevotella*, *Parvimonas*, and some *Streptococcus* species (for example, *S. constellatus*). The orange complex is associated with the transition from health to disease due to a change in environmental conditions that favours colonization by *Porphyromonas gingivalis*, *Tannerella forsythia*, and *Treponema denticola*, which are red-complex species that are considered the main pathogens in periodontal disease ( Socransky *et al*., 1988).

Propolis is a resinous mixture obtained by bees from tree buds, sap exudates or other plant sources and then processed in the hive as a sealant for small holes ( Bueno-Silva *et al*., 2017; Santos *et al*., 2019). Propolis has been considered a promising source of bioactive molecules with pharmacological properties, including antimicrobial ( Alencar *et al*., 2007; Bueno-Silva *et al*., 2013, 2015, 2016; Dantas Silva *et al*., 2017), anti-inflammatory ( Markiewicz-Żukowska *et al*., 2012; Trusheva *et al*., 2004), antioxidant, and antiparasitic activities ( Bueno-Silva *et al*., 2015). Propolis can have different geographical origins ( Freires *et al*., 2016; Lotti *et al*., 2010; Piccinelli *et al*., 2011), and although they may share similar physical characteristics, the chemical compositions of propolis isolates from different sites cannot be the same because the composition depends on the place, time, method of collection, and plants of origin ( Becerra *et al*., 2019). The chemical composition of propolis is closely related to the resins and balsams of the plant sources used to produce it. More than 300 chemical components of propolis have been identified ( Barbosa Bezerra et al., 2017; Bryan *et al*., 2016), and the main groups are waxes, polyphenols (phenolic acids, flavonoids), and terpenoids ( Bonamigo *et al*., 2017; De Freitas *et al*., 2018; El-Guendouz *et al*., 2018; Hochheim *et al*., 2020; Popova *et al*., 2015; Svečnjak *et al*., 2020; Touzani *et al*., 2019; Zhang *et al*., 2017).

Some research studies have demonstrated an effect of propolis on oral bacteria biofilms. Veloz *et al*. (2019a) compared some biological properties of individual compounds and mixtures of these compounds from Chilean propolis, such as their antimicrobial and antibiofilm activities against *S. mutans* cultures. Their results suggest that the polyphenols pinocembrin and apigenin exhibit antimicrobial activity against *S. mutans* at low concentrations. Martins *et al*. (2019) evaluated the antibacterial activity, antibiotic effect, and cytotoxic potential of mouthwashes containing Brazilian red propolis *in vitro.* The results showed that red propolis mouthwashes decreased viable cells to 44% after 1 minute of contact with fibroblasts. Thus, red propolis extract was concluded to exhibit antibacterial activity against *Streptococcus* spp. and *Lactobacillus casei* and had cytotoxic and antibiofilm effects similar to those of chlorhexidine.

The diverse chemical compositions of propolis reflect its advantage as an antibacterial agent ( Abbasi *et al*., 2018; Almuhayawi, 2020; Asawahame *et al*., 2015; Cardoso *et al*., 2016; Chen *et al*., 2018; Lupatini *et al*., 2016; Martins *et al*., 2018; Oryan *et al*., 2018; Pasupuleti *et al*., 2017; Przybyłek & Karpiński, 2019; Utispan *et al*., 2017; Veiga *et al*., 2017; Veloz *et al*., 2019b; Zabaiou *et al*., 2017). Despite efforts to investigate its pharmacological properties, no studies have evaluated the action of Peruvian propolis on bacteria present in periodontal biofilms. Therefore, this study aimed to evaluate the antibacterial, antibiofilm, and cytotoxic activities and chemical compositions of Peruvian propolis in an *in vitro* oral biofilm of *S. gordonii* and *F. nucleatum.*

## Methods

### Study design

The experimental work involved a consecutive, *in vitro*, longitudinal, and double-blinded study design. The decision criteria method was used for the present investigation ( Andersen *et al.,* 2019; Bentz *et al.,* 2013). Based on the results obtained in each experiment, it was decided to continue the trials only with those propolis that showed antibacterial activity, antibiofilm and were not cytotoxic to gingival fibroblasts.

### Propolis sampling

The propolis samples were collected from fixed apiaries and wild flora around beehives. The collection process was performed by scraping the boxes with a bevelled plastic spatula and placing them in airtight glass containers covered with dark plastic bags until they were delivered to the Biopolymers and Metallopharmaceuticals Research Laboratory (LIBIPMET) of the National Engineering University, Lima. All propolis were masked by a person not involved in the study and labelled with letters until after data processing was completed. They were then stored refrigerated at 4 °C until processing.

Samples were collected in 13 Andean regions of Peru, considered as static apiaries (they are not mobilized by seasonal changes) ( Table 1).

**Table 1.** List of Peruvian Andean propolis sampling sites.

Sample code	Sampling site	Geographic location
A	Yanasara, La Libertad	10°33′17.8″S 75°25′01.6″W
B	Vitor, Arequipa	16°28′49.6″S 71°50′40.7″W
D	Shiymay, Huánuco	9°57′36.1″S 76°09′29.9″W
E	Chontabamba, Pasco	10°36′04.7″S 75°26′28.7″W
F	San Miguel de Aco, Ancash.	9°22′39.8″S 77°32′33.5″W
G	Saylla, Cusco	13°33′34.7″S 71°50′34.8″W
H	Chanchamayo, Junín	11°08′43.5″S 75°24′26.0″W
I	Caraz, Ancash	9°03′06.9″S 77°49′02.6″W
J	Marcará, Ancash	9°19′37.4″S 77°36′27.9″W
K	La Merced, Junín.	11°04′46.4″S 75°20′28.6″W
L	Yura, Arequipa	16°14′49.4″S 71°42′17.2″W
M	Oxapampa, Pasco	10°39′01.6″S 75°23′14.7″W
N	Siguas, Arequipa	16°20′39.2″S 72°07′38.3″W

### Preparation of the crude extracts of propolis

The 13 samples were removed from refrigeration, and after waiting two hours for them to reach room temperature (RT), they were thinly cut and then macerated in absolute ethanol using a volume of 100 mL per 10 grams of propolis. The samples were then incubated at RT for 24 hours. The macerate was filtered using a glass funnel with a 20-cm diameter and sterile cotton, and the sample was collected in a glass dish that was transferred to an extraction hood for evaporation of the ethanol (until a paste was formed) ( Markiewicz-Żukowska *et al*., 2012).

### Preparation of the liquid–liquid partitions of propolis

The crude extract (CE) was solubilized with a mixture of chloroform (CHCl _3_):methanol (4:1 v:v). Approximately 50 mL of the mixture was needed to completely solubilize each sample. Next, 30 mL of ultrapure water was added to the assembled separation column, followed by solubilized propolis and approximately 250 mL of CHCl _3_. The separation column was left for six hours until both phases were translucent. We added 250 mL of CHCl _3_ and repeated the previous procedure. The chloroform phases were combined in the same beaker. The aqueous phase was recovered in a 100-mL beaker. Both collected phases were taken to the extraction hood for chloroform evaporation ( Eldeniz *et al*., 2007; Santos *et al*., 2002; Verma *et al*., 2018).

In the same assembled separation column as above, 300 mL of 1-butanol (BuOH) was added, and then the aqueous phase obtained above was poured into the column. The formation of two phases (lower aqueous and upper butanolic) was observed. They were allowed to stand for approximately three hours until both phases were completely translucent. Then, both phases were separated and collected in beakers of 500 mL (butanolic phase) and 100 mL (aqueous phase). They were immediately transferred to the extraction hood for the evaporation of the BuOH. Once a paste had formed, 50 mL of water was added. Each sample was frozen at −70 °C for 24 hours and lyophilized twice. The aqueous phase was frozen at −70 °C for 24 hours (in an ultralow-temperature freezer), and then the sample was lyophilized in a 2.5-L LABCONCO freeze-dryer at a pressure of 0.018 mbar and −54 °C. Finally, three residues were obtained for each propolis sample: chloroform residue (RCHCl _3_), butanolic residue (RBuOH), and aqueous residue (RH _2_O) ( Simões *et al*., 2004; Yang *et al*., 2011).

### Obtaining the propolis fractions from the chloroform residue (RCHCl _3_)

After the propolis partitions were obtained, RCHCl _3_ was fractionated using Sephadex LH-20 as the stationary phase and the solvents petroleum ether (PE), dichloromethane (DCM), and methanol (MeOH) as the mobile phase. RCHCl _3_ propolis was first solubilized with PE. Open-column chromatography was performed with Sephadex LH-20 as the stationary phase (100 g) in PE. The solubilized sample was added to the column and eluted with PE (450 mL), followed by DCM (350 mL), and finally with MeOH (500 mL), thus yielding the PE fraction, dichloromethane fraction, and methanolic fraction. The fractions obtained were dried in an extraction hood for 24 hours ( Mancarz *et al*., 2019; Yang *et al*., 2011).

Finally, the three dry fractions were transferred to the Bacteriology Laboratory of the Universidad Peruana Cayetano Heredia for antimicrobial sensitivity tests.

### Bacterial strains

*S. gordonii* strains (ATCC ^®^ 51656™) and *F. nucleatum* (ATCC ^®^ 10953™) were used for the antibacterial susceptibility and antibiofilm assays. The microorganisms were imported directly from the American Type Culture Collection ( ATCC, USA) and cultured in the Bacteriology Laboratory of the Research and Development laboratories, School of Sciences and Philosophy of the Universidad Peruana Cayetano Heredia ( Voos *et al*., 2014).

### Evaluation of antibacterial activity

The disk diffusion method was used for the antimicrobial susceptibility test. Petri dishes containing trypticase soy agar (TSA) were prepared for *S. gordonii* (ATCC ^®^ 51656™). Petri dishes containing TSA supplemented with 5% sheep blood, hemin (5 mg/L), and menadione (1 mg/L) were prepared for *F. nucleatum* (ATCC ^®^ 10953™). The dishes were monitored for 24 hours to confirm their sterility ( Millones-Gómez *et al*., 2020).

The challenge was performed by embedding a swab with the previously prepared inoculum, spreading the inoculum on the surface of the agar four times, allowing it to stand for five minutes, and then placing Whatman No. 3 filter paper discs (6-mm diameter) impregnated with 10 μL of each of the propolis extracts 25 mg/mL on top but away from one another. This procedure was repeated four times to yield plates for each of the extracts (CE, partitions, and fractions). A 0.12% chlorhexidine solution was used as a positive control, and dimethyl sulfoxide (DMSO) and Milli-Q water (1:1) were used as negative controls. The evaluation of antibacterial activity was carried out by means of inhibition zones in millimeters using a Truper calibrator ( Millones-Gómez *et al*., 2020).

### First decision criteria

After evaluating the CEs of the 13 propolis samples, we decided that partitions would only be performed on the extracts that exhibited zones of inhibition on both strains studied, and then we could perform cytotoxic evaluations against human gingival fibroblasts.

### Evaluation of the cytotoxic effect of propolis on cell line HGF-1 (ATCC ^®^ CRL-2014)

Cytotoxicity was evaluated in the dentistry laboratory of the Center for Research and Development in Health Sciences of the Universidad Autónoma de Nuevo León, Mexico. The cytotoxic effect of propolis on human gingival fibroblast cell line (HGF-1 ATCC ^®^ CRL-2014, American Type Culture Collection; USA) was determined by adapting the cell viability method for microplates using the MTT assay as described below ( Devequi-Nunes *et al*., 2018; Poggio *et al*., 2017). Cytotoxicity responses were classified as severe (30%), moderate (30-60%), mild (60-90%), or noncytotoxic (> 90%) ( Poggio *et al*., 2017).

### Second decision criteria

After evaluating cytotoxicity, we decided that only residues that showed cell viability greater than 90% in the HGF-1 cell line (ATCC ^®^ CRL-2014) would be fractionated and evaluated for antimicrobial activity.

### Minimum inhibitory concentration

The method used was microdilution in broth using 96-well microtiter plates. Chlorhexidine at 0.12% was the positive control, and diluted DMSO solution with Milli-Q water (1:1) was the negative control. The entire procedure was repeated four times for each propolis sample with the largest inhibition zones ( Millones-Gómez *et al*., 2020). The minimum inhibitory concentration of propolis was deemed to be the concentration in the well where no development was observed (turbidity) using a spectrophotometer.

### Third decision criteria

After determining the minimum inhibitory concentration of fractions with zones of inhibition on both strains studied, we evaluated the biofilm considering those concentrations with antibacterial and nontoxic effects on the HGF-1 cell line (ATCC ^®^ CRL-2014).

### Formation of a biofilm of *S. gordonii* and *F. nucleatum*

Each strain was inoculated from a colony into 15 mL of Trypticase soy broth and incubated at 37 °C in anaerobiosis until reaching the exponential growth phase of *S. gordonii* (four hours and 30 minutes) and *F. nucleatum* (eight hours), which was shown by an optical density of 550 nm and 0.125 nm with 150 × 10 ^6^ cells/mL ( Sánchez *et al*., 2011).

Subsequently, the surfaces of Thermo Scientific Nunc Lab-Tek Chamber Slides were prepared with 30 μL of poly- l-lysine incubated at RT for 30 minutes and then allowed to dry at 37 °C for 24 hours under sterile conditions. Then, 300 μL of artificial saliva was added for incubation at 4 °C for 16 hours. The sheets were washed with 300 μl of phosphate-buffered saline (PBS 1×) twice. A total of 280 μL of brain heart infusion (BHI) + 2.5% sucrose was added. Finally, 10 μL of *S. gordonii* + 10 μL of *F. nucleatum* was inoculated, and then each slide was incubated for 24 hours at 37 °C under anaerobic conditions ( Sánchez *et al*., 2011).

### Biofilm analysis by PCR

Biofilm analysis by polymerase chain reaction (PCR) was performed in the Bioinformatics and Molecular Biology Laboratory of the Research and Development Laboratories of Universidad Peruana Cayetano Heredia. To confirm the presence of both species, DNA was extracted from the cultured biofilms at 48 and 120 hours. The DNA concentration was quantified in a Thermo Scientific Nanodrop 2000 using Maxima SYBR Green/ROX qPCR Master Mix (2X) (for absolute quantification by real-time PCR using the following cycling conditions: Initial denaturation: 95 °C, 3 min. PCR cycling 45 cycles (95 °C, 15 s, 60 °C, 30 s). Each sample was diluted to 100 ng/μL to determine the proportions of cells of both species ( Sánchez, 2018; Sánchez *et al*., 2011).

*S. gordonii* ( Ayala *et al*., 2016; Cayo *et al*., 2016) and *F. nucleatum* ( Jacinto, 2018; Sánchez, 2018) were identified using specific primers:

*srtA* F 5′ TATTATGGTGCTGGTACGATGAAAGAGACTC 3′;

*srtA* R 5′ TATAGATTTTCATACCAGCCTTAGCACGATC 3′;

*radD* F 5′ GGATTTATCTTTGCTAATTGGGGAAATTATAG 3′; and

*radD* R 5′ ACTATTCCATATTCTCCATAATATTTCCCATTAGA 3′.

### Evaluation of the effect of propolis on the biofilm

To evaluate the effect of propolis on the biofilm, 10 μL of *S. gordonii* + 10 μL of *F. nucleatum* was inoculated, and then each plate was incubated for 24 hours at 37 °C under anaerobic conditions. The propolis fraction with the greatest antibacterial effect and lowest toxicity against HGF-1 cells was used.

The first dose of the propolis extract was administered 24 hours after its formation, where the supernatant was carefully removed from each well. Two washes were performed without exerting much force with 300 μL of PBS (1×). Then, 300 μL of propolis was added, followed by incubation for 1 minute at RT. The plate was removed and washed up to the surface of each well with 300 μL of PBS (1×) twice. Next, 300 μL of sterile BHI + SAC 2.5% culture medium was added, and the plate was incubated at 37 °C for 24 hours under anaerobic conditions ( Sánchez *et al*., 2011).

The following groups were formed:

- Group 1 (Sg + Fn): 300 μL of propolis was pipetted in each well every 24 hours until evaluation at two days and five days.
- Group 2 (negative control): 300 μL DMSO + Milli-Q water (1:1) was pipetted in each well every 24 hours until evaluation at two days and five days.
- Group 3 (positive control): 300 μL of 0.12% chlorhexidine was pipetted in each well every 24 hours until evaluation at two days and five days.
- Three repetitions of each group were performed.

The final exposure of the slide occurred when the biofilms reached 120 hours of maturation, where the Nunc Lab-Tek Chamber Slide™ system fixation and staining procedure was performed with fluorochromes for confocal microscopy.

### Fixation and staining of slides

The removable chamber was taken off the surface of the Lab-Tek slide, placed horizontally to fix each surface of each well with 30 μL of 4% paraformaldehyde (4%) that was added over the entire surface of the biofilm, and incubated RT for 20 minutes. Then, three gentle washes were performed with 1× PBS. Five microlitres of the L13152 LIVE/DEAD ^®^ BacLight bacterial viability kit was added at a concentration ratio of 1:1 with fluorochromes to the surface of the biofilm. The cells were stained with 5 μL of VECTASHIELD ^®^ HardSet™ Antifade Mounting Medium with DAPI (4′,6-diamidino-2-phenylindole).

H-1500-10.This last step was performed to differentiate the nucleic acids (DNA) in the bacteria and conserve the samples ( Sánchez *et al*., 2011).

### Measurement of biofilm thickness

**Figure 1.**
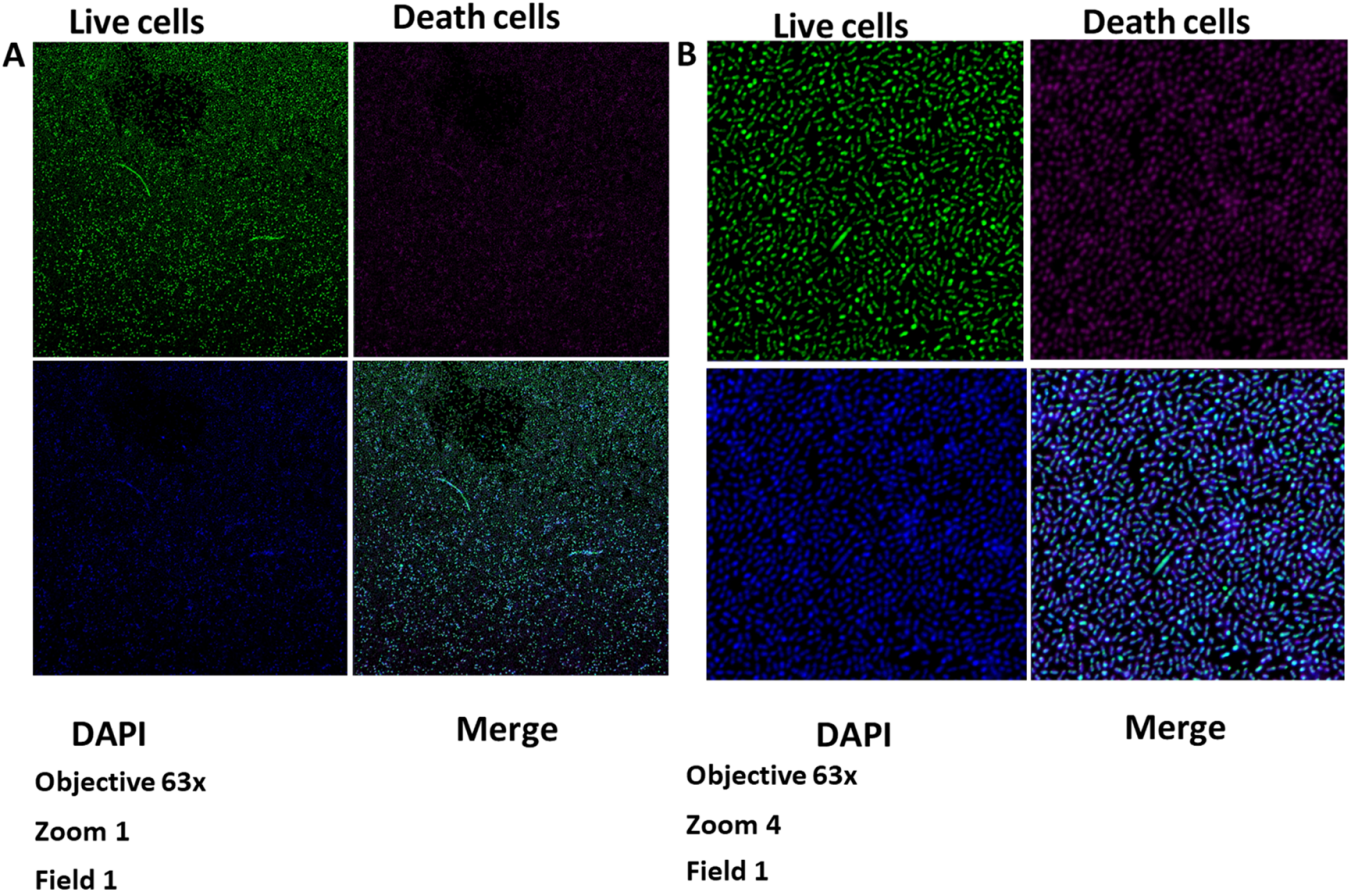
Growth of the biofilm of *S. gordonii* (ATCC ^®^ 51656™) and *F. nucleatum* (ATCC ^®^10953™) at 48 hours. Images taken by confocal microscopy (Zeiss LSM 880). A) Zoom 1. B) Zoom 4. DAPI, 4′,6-diamidino-2-phenylindole.

**Figure 2.**
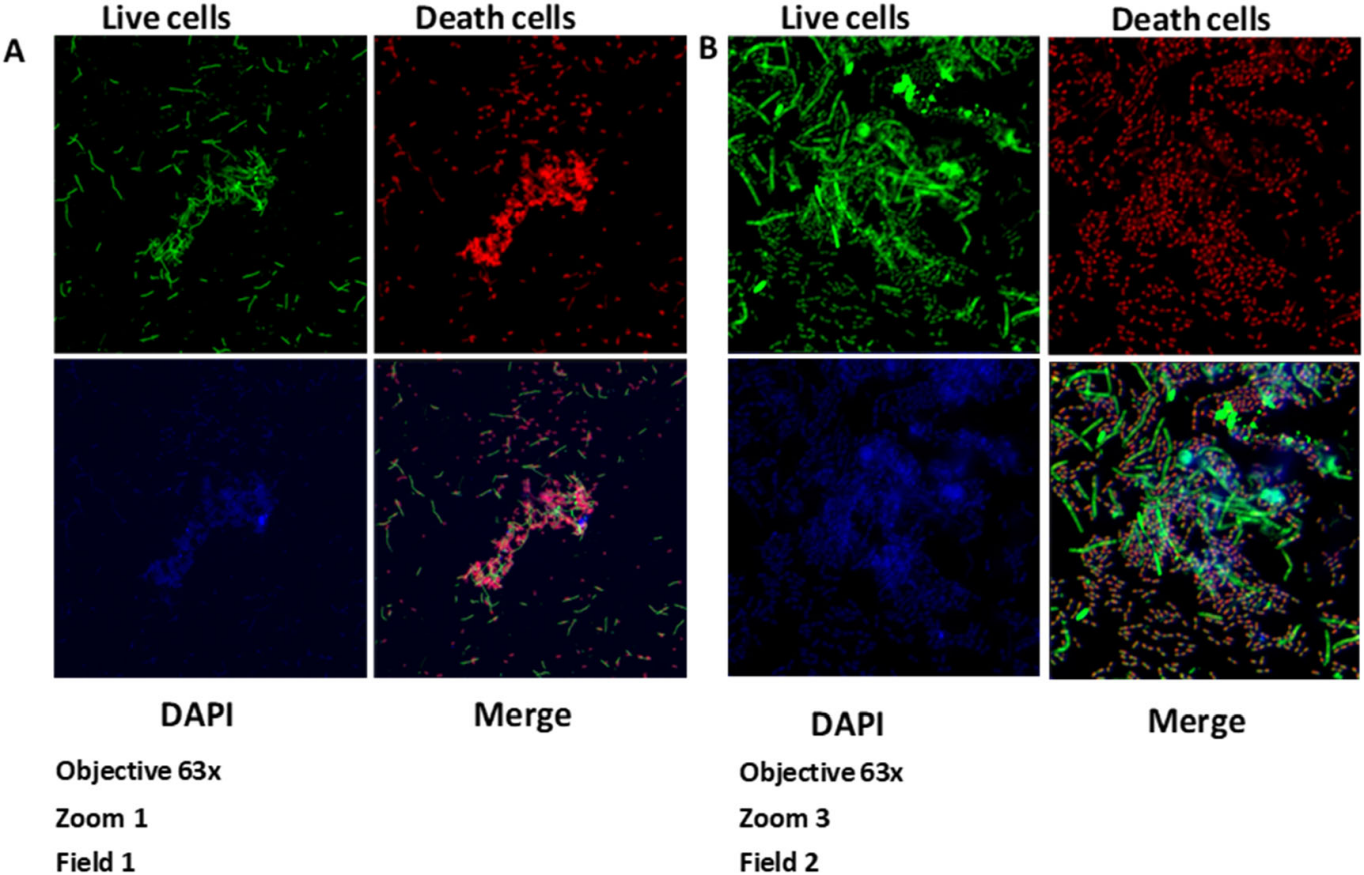
Growth of the biofilm of *S. gordonii* (ATCC ^®^ 51656™) and *F. nucleatum* (ATCC ^®^10953™) at 120 hours. Images taken by confocal microscopy (Zeiss LSM 880). A) Zoom 1. B) Zoom 3. DAPI, 4′,6-diamidino-2-phenylindole.

**Figure 3.**
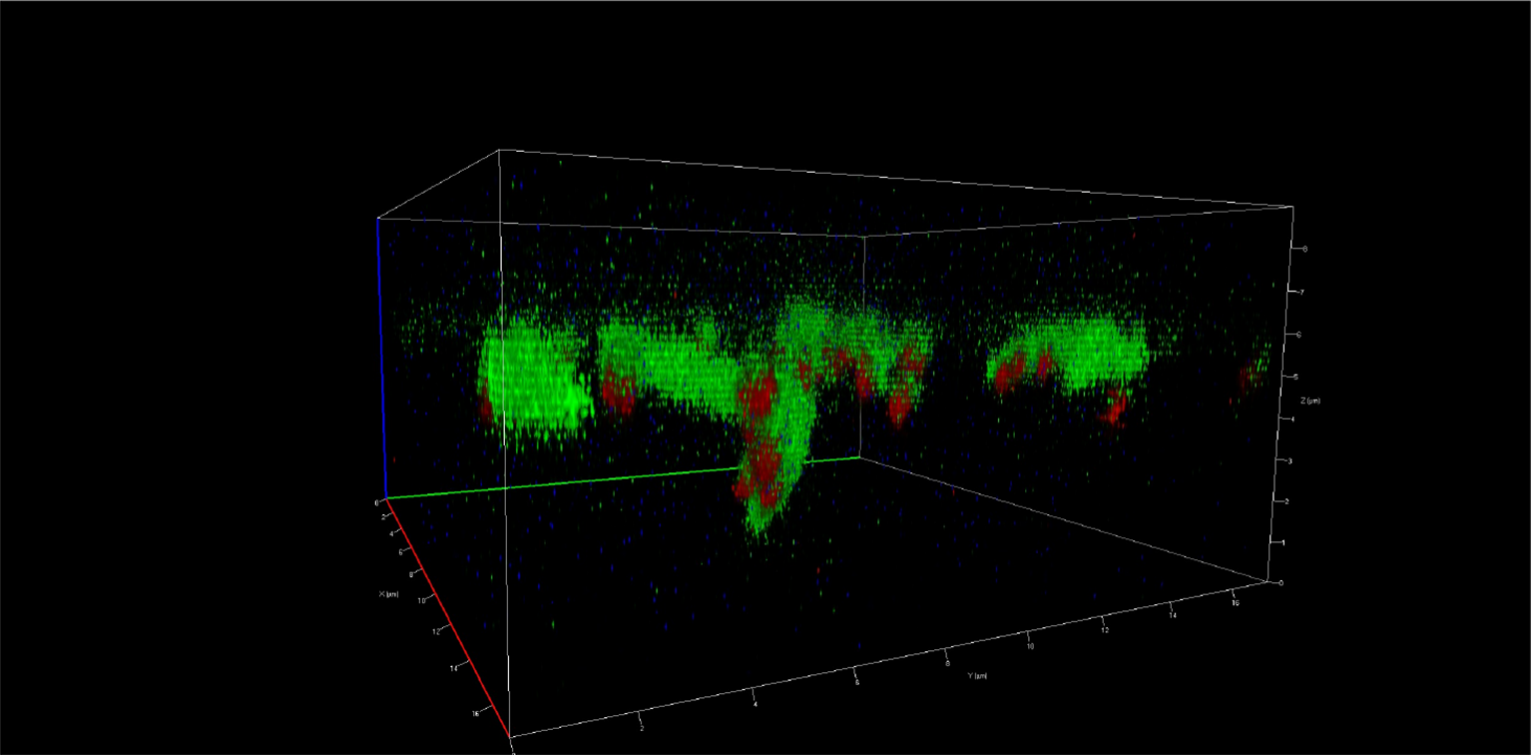
Vertical growth of the biofilm of *S. gordonii* (ATCC ^®^ 51656™) and *F. nucleatum* (ATCC ^®^ 10953™) at 120 hours. Images taken by confocal microscopy (Zeiss LSM 880).

Biofilm thickness was evaluated in the Microscope Core, Neuroscience Department, School of Medicine, University of California San Diego - USA using a Zeiss LSM880 confocal microscope. Biofilm thickness (vertical growth) was measured with an LSM880 confocal microscope (Zeiss) using the Z-Stacks option (3D images). The images were captured with the Zeiss Plan-Apochromat objective of 63×/1.4 oil objective, with two different zooms. The resulting images were processed using Zeiss Zen Black software (version 2012), and the results were read in micrometres ( Figures 1A, 1B, 2A, 2B, and 3).

### Chemical composition of propolis

**Figure 4.**
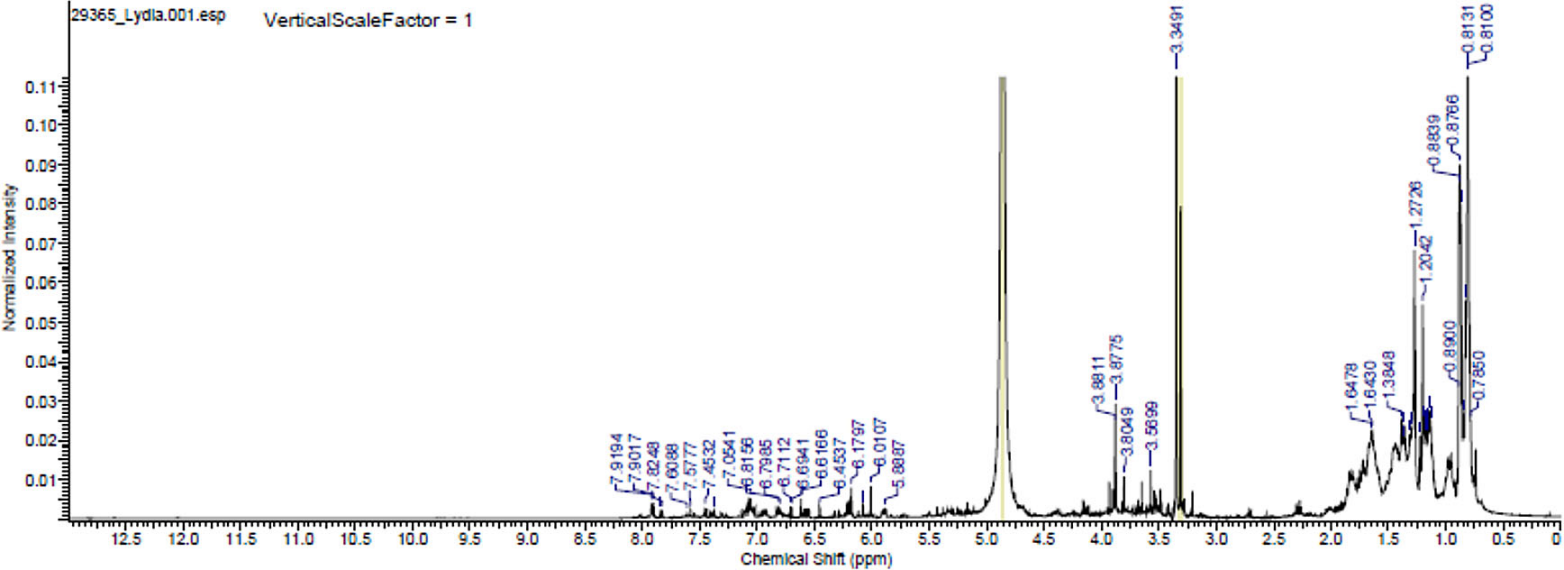
Proton nuclear magnetic resonance (NMR) spectrum (500 MHz, CD _3_OD, *ppm*) of the methanolic fraction of Oxapampa propolis.

**Figure 5.**
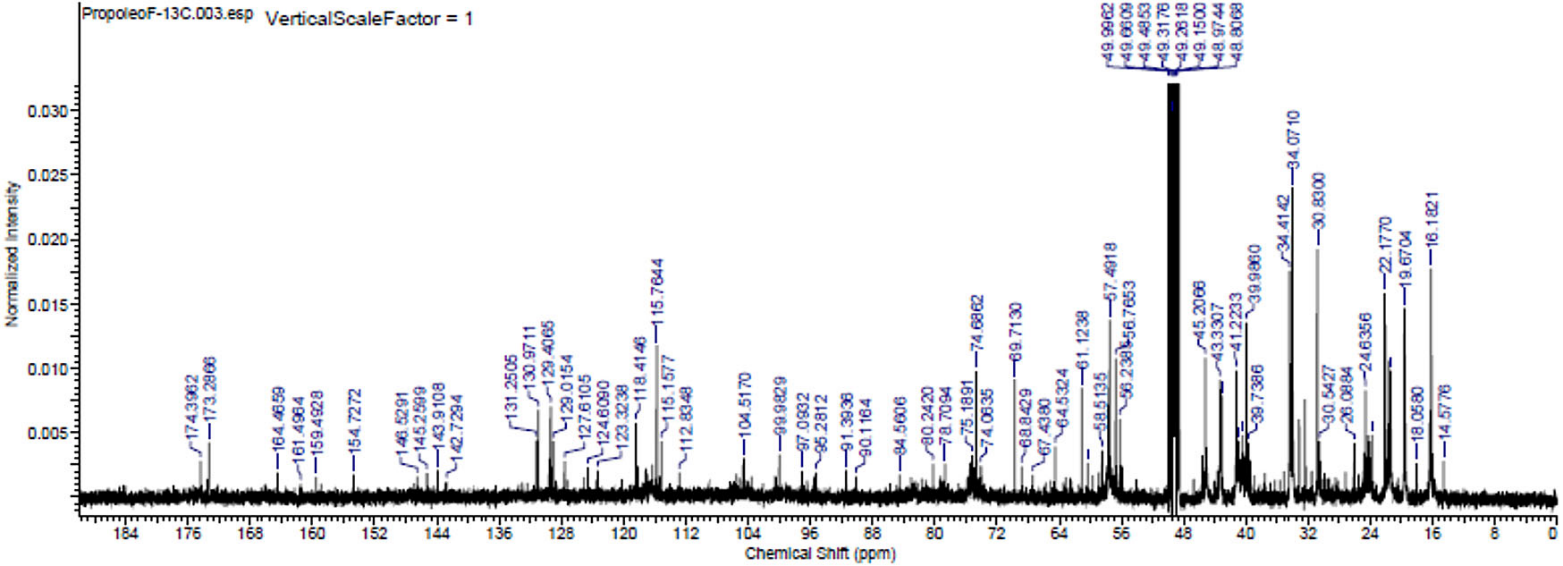
Carbon-13 nuclear magnetic resonance spectrum (125 MHz, CD _3_OD, *ppm*) of the methanolic fraction of Oxapampa propolis.

The major chemical components of the propolis fraction with effects on the biofilm were analysed through proton- and C-13 nuclear magnetic resonance ( ^1^H NMR and ^13^C NMR) at the Analytical Center of the Institute of Chemistry of the University of São Paulo, Brazil. A 25-mg sample was solubilized in MERCK brand deuterated methanol, and NMR spectra were recorded in a 500-MHz Bruker spectrometer operating at 500 MHz to obtain the hydrogen spectra and operating at 125 MHz for the C-13 spectra ( Figures 4 and 5).

**Figure 6.**
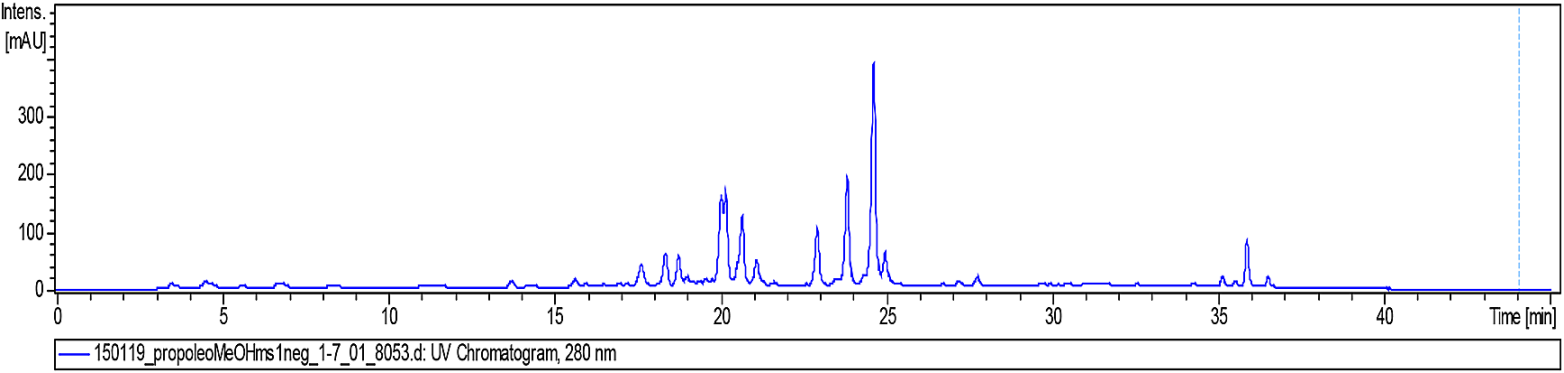
Analysis of the methanolic fraction of Oxapampa propolis by liquid chromatography coupled to mass spectrometry in positive mode.

Then, a 2-mg sample of FMeOH from RCHCl _3_ of Oxapampa propolis was solubilized in methanol (CH3OH), filtered through a filter with a pore size of 0.45 μm, and transferred to a 2-mL vial. The sample was analysed by liquid chromatography coupled to mass spectrometry (LC/MS) at the Laboratory of Natural Products of the Institute of Chemistry of the University of São Paulo, Brazil. The liquid chromatographer was of the Shimadzu brand (Kyoto, Japan), which consists of two LC-20AD model analytical pumps, with an SIL-20AHT automatic injector and an SPD-20A UV/Vis detector, an oven for the CTO-20A column, and a CBM-20A controller. The mobile phase consisted of two solvents: solvent A: 0.2% formic acid in water and solvent B: acetonitrile (ACN). The gradient used was 5% B T0-2 minutes, and for T2-35 minutes, a gradient up to 100% B, T35-45 minutes was maintained at 100% B. The column was a Luna 5u PFP (2) 100 A, 150 × 2 mm (Phenomenex). The analyses were performed at 254 nm and 280 nm, the oven for the column was set at 40 °C, and the flow of the mobile phase to the mass spectrometer was 200 μL/min ( Figure 6).

**Figure 7.**
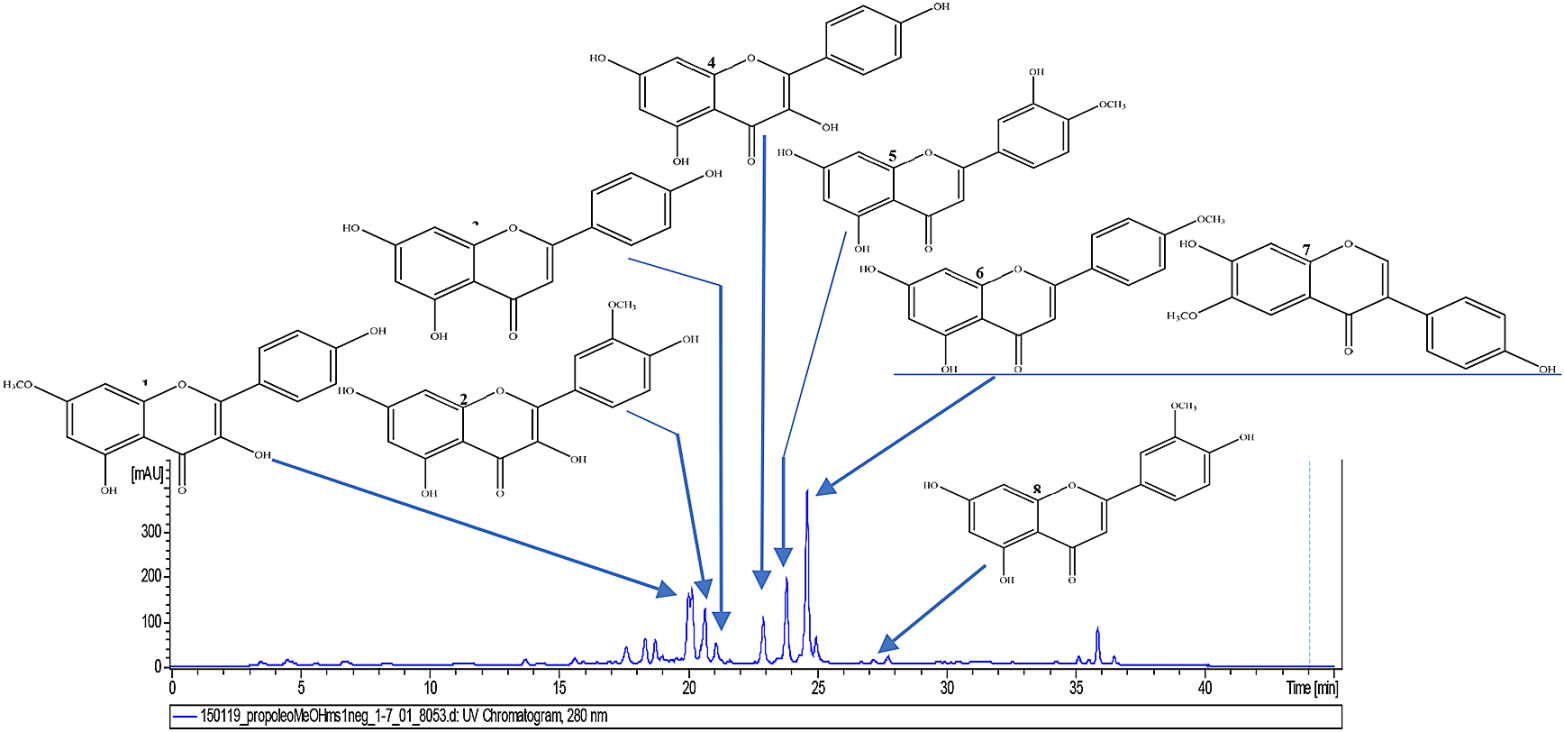
Chromatogram (UV, λ 280 nm) of the methanolic fraction of Oxapampa propolis with the identified components rhamnocitrin (1), isorhamnetin (2), apigenin (3), kaempferol (4), diosmetin (5), acacetin (6), glycitein (7), and chrysoeriol (8).

The mass spectrometer (Bruker MicroTOF-QII) operated in positive MS/MS mode with N _2_ gas for nebulization and drying at 4 bar and 8 L/min, respectively. The drying temperature was programmed at 200 °C, the collision and quadrupole energies were 10 and 8 eV, respectively, and the capillary received a charge of 4,500 V. Cones RF1 and RF2 were programmed at 400 and 200 Vpp, respectively, and the hexapole RF and collision RF were both set at 200 Vpp. The mass reading range was 100 to 1,000 kDa ( Narimane *et al*., 2020; Pobiega *et al*., 2019; Sun *et al*., 2015) ( Figure 7).

### Statistical analysis

For the analysis, the statistical software SPSS version 25.0 was used. The Kruskal-Wallis test was used to compare the average range of each treatment with the average range of all of them. The Z test was used to individually compare the average range of each type of treatment to the average range of all treatments. Dunn’s multiple comparisons test was used for decision processes in sequential experiments.

## Results

The objective of this study was to evaluate the antibacterial effect of the CEs of 13 propolis isolates from the Peruvian Andes ( Table 2). The microscopy images and raw data are available in *Underlying data* ( Millones-Gómez, 2021). The Kruskal-Wallis H test indicated a difference between some of the experimental and/or control propolis in their growth inhibition of both *S. gordonii* (p < 0.001) and *F. nucleatum* (p < 0.001). Likewise, the Z test revealed that, together with chlorhexidine ( *S. gordonii*: 14.45 ± 0.30; *F. nucleatum*: 14.40 ± 0.18 mm), the propolis that exceeded the average inhibition in both types of bacteria were those from Oxapampa (10.88 ± 0.10 and 10.88 ± 0.10 mm), Chontabamba (10.0 ± 0.16 and 10.05 ± 0.19 mm), Saylla (10.00 ± 0.18 and 10.00 ± 0.14 mm), and Yura (9.98 ± 0.17 and 10.00 ± 0.16 mm). When performing post hoc comparisons of propolis with chlorhexidine, the difference in their average ranges was not statistically significant; thus, they are listed in Table 2 with the same letter.

**Table 2.** Antibacterial effects of crude extracts of 13 Peruvian propolis isolates on strains of *S. gordonii* and *F. nucleatum.*

	Zones of inhibition (mm)							
	*S. gordonii* ^1^				*F. nucleatum* ^1^			
	Mean (SD)	Range ^2^ ^,^ ^3^			Mean (SD)	Range ^2^ ^,^ ^3^		
Yanasara	6.28(±0.15)	35.9	ns		7.65(±0.10)	34.5	ns	
Vitor	6.28(±0.15)	35.9	ns		8.20(±0.16)	38.5	ns	
Shiymay	0.00(±0.00)	11	++		0.00(±0.00)	16.5	+	
Chontabamba	10.00(±0.16)	46.8	*	a	10.05(±0.19)	47.4	*	a
San Miguel de Aco	0.00(±0.00)	11	ns		0.00(±0.00)	16.5	ns	
Saylla	10.00(±0.18)	46.6	*	a	10.00(±0.14)	46.1	*	a
Chanchamayo	6.10(±0.14)	31.8	ns		0.00(±0.00)	16.5	+	
Caraz	0.00(±0.00)	11	++		0.00(±0.00)	16.5	+	
Marcará	1.73(±0.15)	20.8	ns		0.00(±0.00)	16.5	+	
La Merced	2.58(±0.35)	25.8	ns		0.00(±0.00)	16.5	+	
Yura	9.98(±0.17)	46.1	*	a	10.00(±0.16)	46	*	a
Oxapampa	10.88(±0.09)	54.5	**	a	10.88(±0.10)	54.5	**	a
Siguas	0.00(±0.00)	11	ns		0.00(±0.00)	16.5	+	
Chlorhexidine 0.12%	14.45(±0.30)	58.5	***	a	14.40(±0.18)	58.5	***	a
Dimethyl sulfoxide + water (1:1)	0.00(±0.00)	11	++		0.00(±0.00)	16.5	+	
Overall		30.5				30.5		

^1^ Kruskal-Wallis H, *S. gordonii* (p = 0.000 < 0.001) and *F. nucleatum* (p = 0.000 < 0.001), pairwise comparisons.

^2^ Z-test for p-values (<0.05, <0.01, and <0.001): *,**, or *** indicates greater than the midrange, and +, ++, or +++ indicates less than the midrange; ns: not significant.

^3^ Dunn test for multiple comparisons, where the same letter indicates no difference between treatments exceeding the average.

When evaluating the CE partitions with the greatest antibacterial effect ( Table 3), the Kruskal-Wallis H test indicated a difference between some of the experimental propolis partitions and/or controls in the growth inhibition of both *S. gordonii* (p < 0.001) and *F. nucleatum* (p < 0.001). Likewise, the Z test revealed that, together with chlorhexidine ( *S. gordonii*: 14.45 ± 0.30 and *F. nucleatum*: 14.40 ± 0.18 mm), the partition that exceeded the average inhibition in both types of bacteria was the chloroform residue from Oxapampa (9.40 ± 0.28 and 9.95 ± 0.19 mm). The chloroform residues from Saylla (10.2 ± 0.14 mm) and Yura (9.27 ± 0.05 mm) showed above-average *S. gordonii* inhibition. The butanolic residues from Saylla (9.98 ± 0.21 mm) and Oxapampa (9.45 ± 0.19 mm) showed above-average inhibition of *F. nucleatum.* When performing post hoc comparisons of the best propolis with chlorhexidine, the difference in their average ranges was not statistically significant; thus, they are written in Table 3 with the same letter.

**Table 3.** Antibacterial effects of the partitions of crude extracts of Peruvian propolis showing antibacterial activity against *S. gordonii* and *F. nucleatum.*

		Zones of inhibition (mm)							
		*S. gordonii* ^1^				*F. nucleatum* ^1^			
Propolis	Partition	Mean (SD)	Range ^2^ ^,^ ^3^			Mean (SD)	Range ^2^ ^,^ ^3^		
Chontabamba	Aqueous residue	0.00(±0.00)	12.5	+		0.00(±0.00)	10.5	++	
	Chloroform residue	9.90(±0.48)	45.1	**	a	9.20(±0.16)	37.9	ns	
	Butanolic residue	0.00(±0.00)	12.5	+		8.60(±0.16)	30	ns	
Saylla	Aqueous residue	0.00(±0.00)	12.5	+		0.00(±0.00)	10.5	++	
	Chloroform residue	10.20(±0.14)	49.5	**	a	8.40(±0.00)	27	ns	
	Butanolic residue	8.80(±0.16)	27	ns		9.98(±0.21)	48.6	**	a
Yura	Aqueous residue	0.00(±0.00)	12.5	+		0.00(±0.00)	10.5	++	
	Chloroform residue	9.28(±0.05)	39.9	*	a	8.20(±0.00)	22.5	ns	
	Butanolic residue	9.30(±0.48)	35.6	ns		9.10(±0.12)	36	ns	
Oxapampa	Aqueous residue	0.00(±0.00)	12.5	+		0.00(±0.00)	10.5	++	
	Chloroform residue	9.40(±0.28)	40	*	a	9.95(±0.19)	48.4	**	a
	Butanolic residue	9.08(±0.10)	32.4	ns		9.45(±0.19)	41.6	*	a
Chlorhexidine 0.12%		14.45(±0.30)	54.5	***	a	14.40(±0.18)	54.5	***	a
Dimethyl sulfoxide + water (1:1)		0.00(±0.00)	12.5	+		0.00(±0.00)	10.5	++	
	Overall		28.5				28.5		

^1^ Kruskal-Wallis H, *S. gordonii* (p = 0.000 < 0.001) and *F. nucleatum* (p = 0.000 < 0.001), pairwise comparisons.

^2^ Z-test for p-values (<0.05, <0.01, and <0.001): *,**, or *** indicates greater than the midrange, and +, ++, or +++ indicates less than the midrange; ns: not significant.

^3^ Dunn test for multiple comparisons, where the same letter indicates no difference between treatments exceeding the average.

When evaluating the cytotoxicity of the propolis partitions on human gingival fibroblast cell line (HGF-1 ATCC ^®^ CRL-2014, American Type Culture Collection; USA) considering 10% Triton X-100 (negative control) and the medium (positive control) ( Table 4), we generally observed that increasing the dilution decreased the cell viability of the partitions of the propolis evaluated. The chloroform partition of Oxapampa propolis showed its highest percentage of cell viability (92.64%) at the 0.78 mg/mL dilution.

**Table 4.** Cytotoxicity of the propolis partitions with greater antibacterial effects on *S. gordonii* and *F. nucleatum* in the human gingival fibroblast cell line.

Cell viability (%)										
Dilutions mg/mL	Medium	Triton X-100 10%	Chontabamba chloroform	Chontabamba butanolic	Saylla chloroform	Saylla butanolic	Yura chloroform	Yura butanolic	Oxapampa chloroform	Oxapampa butanolic
**50**	100	0.815	41.351	14.799	12.279	1.913	2.019	2.3	52.657	2.386
**25**	100	0.815	45.402	17.665	13.275	2.945	3.432	3.565	64.892	3.516
**12.5**	100	0.815	51.871	22.049	14.685	4.374	4.639	4.9	76.912	4.701
**6.25**	100	0.815	64.586	27.1	16.759	7.178	7.12	6.969	78.735	6.981
**3.125**	100	0.815	71.462	31.962	19.266	9.018	9.153	9.372	85.41	9.154
**1.563**	100	0.815	80.581	37.652	21.285	11.065	11.289	11.727	90.931	11.288
**0.781**	100	0.815	84.327	46.334	23.325	13.223	13.42	14.601	92.641	14.478
**0.39**	100	0.815	85.852	55.664	24.71	14.52	12.045	15.101	93.905	21.212
**0.195**	100	0.815	88.557	71.185	36.872	19.992	16.333	23.165	94.956	23.939
**0.098**	100	0.815	91.667	78.38	58.387	29.697	24.874	35.356	96.52	36.05
**0.049**	100	0.815	95.817	83.439	83.297	42.095	35.41	46.607	96.973	53.907
**0.024**	100	0.815	98.845	89.856	98.856	80.69	55.147	59.85	97.323	88.647

When evaluating the antibacterial effects of the fractions of the chloroform partition of Oxapampa propolis ( Table 5) by the Kruskal-Wallis H test, a difference was identified in the inhibition of *S. gordonii* (p < 0.01) and *F. nucleatum* (p < 0.01) prepared in the different dilutions. The methanolic fraction of the chloroform residue of Oxapampa propolis (12.55 ± 0.19 and 12.15 ± 0.19 mm) had greater inhibition power against each bacterium than when combined with chlorhexidine (14.55 ± 0.19 and 14.33 ± 0.19 mm), exceeding the average. In addition, post hoc analysis revealed that both fractions did not differ from each other but had greater inhibitory power towards both bacteria.

**Table 5.** Antibacterial effects of selected propolis fractions against *S. gordonii* and *F. nucleatum.*

		Zones of inhibition (mm)							
		*S. gordonii* ^1^				*F. nucleatum* ^1^			
Propolis	Fraction	Mean (SD)	Range ^2^ ^,^ ^3^			Mean (SD)	Range ^2^ ^,^ ^3^		
Oxapampa	F. of petroleum ether	0.00(±0.00)	6.5	+	b	0.00(±0.00)	6.5	+	b
	F. of dichloromethane	0.00(±0.00)	6.5	+	b	0.00(±0.00)	6.5	+	b
	F. methanolic	12.55(±0.10)	14.5	*	a	12.15(±0.19)	14.5	*	a
Chlorhexidine 0.12%		14.55(±0.19)	18.5	***	a	14.33(±0.19)	18.5	***	a
Dimethyl sulfoxide + water (1:1)		0.00(±0.00)	6.5	+	b	0.00(±0.00)	6.5	+	b
	Overall		10.5				10.5		

^1^ Kruskal-Wallis H, *S. gordonii* (p = 0.000 < 0.001) and *F. nucleatum* (p = 0.000 < 0.001), pairwise comparisons.

^2^ Z-test for p-values (<0.05, <0.01, and <0.001): *,**, or *** indicates greater than the midrange, and +, ++, or +++ indicates less than the midrange; ns: not significant.

^3^ Dunn test for multiple comparisons, where the same letter indicates no difference between treatments exceeding the average.

When evaluating the concentrations of 0.781 and 1.563 mg/mL of the methanolic fraction of the chloroform residue of Oxapampa propolis on biofilm thickness by confocal microscopy, the Kruskal-Wallis H test indicated that there was a difference in the thickness of the biofilm formed with the different treatments, both at 48 hours (p < 0.01) and at 120 hours (p < 0.001). The biofilm formed with 0.12% chlorhexidine (8,100 ± 1,149 and 45,200 ± 5,718 μm) was not thinner than the average, as shown in Table 6, at either 48 hours or 120 hours (p > 0.05). The biofilms with lower-than-average thickness were those formed with 0.78 mg/μL propolis (8.026 ± 1.609 and 6.840 ± 1.675 μm) and 1.56 mg/μL propolis (7.374 ± 1.620 and 9.248 ± 0.679 μm). Post hoc tests indicated that these did not differ from chlorhexidine in the biofilm formed at 48 hours; but at 120 hours, although they did not differ from each other, propolis made with a concentration of 0.78 mg/μL was thinner than that made with chlorhexidine.

**Table 6.** Effect on biofilm thickness by confocal microscopy of the propolis fractions with antibacterial effects on *S. gordonii* and *F. nucleatum* and nontoxicity in the human gingival fibroblast cell line.

Concentration	Biofilm thickness (μm) at 48 hours ^1^				Biofilm thickness (μm) at 120 hours ^1^			
	Mean (SD)	Range ^2^ ^,^ ^3^			Mean (SD)	Range ^2^ ^,^ ^3^		
0.78 mg/μL propolis	8.03(±1.61)	9.1	+	A	6.84(±1.68)	3.3	+++	A
1.56 mg/μL propolis	7.37(±1.62)	5.6	++	A	9.24(±0.68)	7.7	+	AB
Chlorhexidine 0.12%	8.10(±1.15)	9.3	ns	A	45.20(±5.72)	13.2	ns	BC
Dimethyl sulfoxide + water (1:1)	14.32(±1.82)	21.6	***	B	62.40(±4.39)	20.7	**	C
Without treatment	13.61(±0.81)	19.4	**	B	61.60(±5.51)	20.1	**	C
Overall		13				13		

^1^ Kruskal-Wallis H, *S. gordonii* (p = 0.000 < 0.001) and *F. nucleatum* (p = 0.000 < 0.001), pairwise comparisons.

^2^ Z-test for p-values (<0.05, <0.01, and <0.001): *,**, or *** indicates greater than the midrange, and +, ++, or +++ indicates less than the midrange; ns: not significant.

^3^ Dunn test for multiple comparisons, where the same letter indicates no difference between treatments exceeding the average.

When evaluating the effect of the methanolic fraction of the chloroform residue of Oxapampa propolis on the number of bacteria by quantifying the number of copies of the *srtA* gene of *S. gordonii* and the *radD* gene of *F. nucleatum* in the biofilm by PCR, the Kruskal-Wallis H test indicated that there was a difference in the number of *S. gordonii* (p < 0.01, in both periods) and in the number of *F. nucleatum* (p < 0.05 in both periods) under the different treatments. The concentration of propolis of 1.56 mg/mL decreased the amount of *S. gordonii* both at 48 (6.347 ± 0.410) and at 120 hours (5.157 ± 0.202) and the amount of *F. nucleatum* at 120 hours (5.747 ± 0.618) compared to the average, as did 0.12% chlorhexidine. However, the post hoc analysis indicated that this concentration showed no difference from chlorhexidine in the control of the development of both bacteria in the times evaluated ( Table 7).

**Table 7.** Evaluation of the propolis fractions with antibacterial effects against *S. gordonii* and *F. nucleatum* according to the amount of bacteria through quantification of the number of copies of the *srtA* Gene of *S. gordonii* and the number of copies of the *radD* gene in *F. nucleatum* in biofilms by polymerase chain reaction.

Time	Concentration	*S. gordonii* ^1^				*F. nucleatum* ^1^			
		(Log _10_ cells)				(Log _10_ cells)			
		Average	Range ^2^ ^,^ ^3^			Average	Range ^2^ ^,^ ^3^		
48 hours	0.78 mg/mL propolis	7.29(±0.21)	8.0	ns	ABC	7.05(±0.20)	9.2	ns	B
	1.56 mg/mL propolis	6.35(±0.41)	4.7	+	AB	6.70(±0.54)	6.8	ns	AB
	Chlorhexidine 0.12%	5.63(±0.42)	2.3	++	A	5.75(±0.00)	2.0	++	A
	Dimethyl sulfoxide + water (1:1)	8.13(±0.41)	11.0	ns	BC	7.17(±0.00)	11.0	ns	BC
	Without treatment	10.14(±0.41)	14.0	ns	C	7.17(±0.00)	11.0	ns	BC
120 hours	0.78 mg/mL propolis	6.46(±0.35)	8.0	ns	ABC	6.34(±0.54)	6.8	ns	ABC
	1.56 mg/mL propolis	5.16(±0.20)	2.2	++	A	5.75(±0.62)	3.5	++	A
	Chlorhexidine 0.12%	5.63(±0.21)	4.8	+	AB	5.87(±0.21)	4.7	+	AB
	Dimethyl sulfoxide+ water (1:1)	8.12(±0.83)	11.0	ns	BC	7.77(±0.21)	11.0	ns	BC
	Without treatment	11.09(±0.36)	14.0	ns	C	8.36(±0.21)	14.0	ns	C
	Overall		8.0				8.0		

^1^ Kruskal-Wallis H, *S. gordonii* (p = 0.000 < 0.001) and *F. nucleatum* (p = 0.000 < 0.001), pairwise comparisons.

^2^ Z-test for p-values (<0.05, <0.01, and <0.001): *,**, or *** indicates greater than the midrange, and +, ++, or +++ indicates less than the midrange; ns: not significant.

^3^ Dunn test for multiple comparisons, where the same letter indicates no difference between treatments exceeding the average.

## Discussion

The present study aimed to evaluate the antibacterial effect of CEs, partitions, and fractions of Peruvian propolis and the effects of fractions on the thickness of *in vitro* biofilms of *S. gordonii* and *F. nucleatum.* Of the 13 CEs of propolis evaluated, only four showed antibacterial effects on both strains. Moreover, the methanolic fraction of Oxapampa propolis showed an effect on the thickness of the biofilm created *in vitro.*

The results of the present investigation showed that of the 13 Peruvian propolis isolates evaluated, four showed antibacterial effects on both bacterial strains. These results differ from those of other investigations that have shown the strong antibacterial power of propolis against all evaluated microorganisms ( Becerra *et al*., 2019; Bueno-Silva *et al*., 2013; Dantas Silva *et al*., 2017; Trusheva *et al*., 2004). Although several studies of Peruvian propolis have shown effects on oral bacteria, all of them focused on other types of oral bacteria. Examples include the La Libertad propolis against *Streptococcus mutans* ( Becerra *et al*., 2019; Sánchez, 2018); Huacho and Cajabamba propolis against *Streptococcus mutans* ( Ayala *et al*., 2016; Cayo *et al*., 2016); and propolis from Arequipa, Huánuco, and Huaraz against *Streptococcus mutans* and *Streptococcus oralis* ( Jacinto, 2018; Vargas, 2019). This study is the first to evaluate the effects of Peruvian propolis on bacteria linked to the growth of subgingival biofilms.

The CEs of propolis with the largest zones of inhibition were those from apiaries in Chontabamba, Saylla, Yura, and Oxapampa, with the last being widely studied and showing similar effects in previous studies ( Alemán *et al*., 2018; Eguizábal & Nakata, 2014; Tovalino & Contreras, 2010). The boundless vegetation of the Oxapampa valley, where species such as *Dictyocaryum lamarckianum* (palm tree), *Juglans neotropica* (Andean walnut), *Cyrtocymura scorpioides*, *Vernonanthura patens (*Kunth), *Baccharis latifolia*, *Cecropia* spp., *Inga* spp., and *Pinus tecunumanii* (timber tree), combined with the tropical climate of this area ( Muñoz & Del Pilar, 2015), offer a variety of active components that are transported by bees to their hives in the form of propolis ( Fernández-Calderón *et al*., 2020; Okińczyc *et al*., 2020; Rivera & Mesía, 2009). This variety enhances their biological properties.

In the present study, after identifying the CEs of the four propolis with the greatest zones of inhibition on each bacterium evaluated, partitions were performed, and the strongest antibacterial effects were found in the butanolic and chloroform residues of all the propolis studied. Although there are no studies with partitions of Peruvian propolis, the few studies carried out on propolis from other sources indicate that some solvents have greater affinity for certain components with biological effects ( Flemming *et al*., 2016). For example, the amounts of flavonoids and terpenes in different extracts were found to decrease in the following order: chloroform extract > ethanolic extract > butanolic extract ( Osés *et al*., 2020; Palomino *et al*., 2009).

In Peru, as in many countries, beekeepers prepare by hand the products derived from the hive. Many therapeutic effects have been attributed to propolis ( Narimane *et al*., 2020). However, many of these products could be used without any control, with several of them being toxic for human consumption; therefore, the toxicity must be correctly recognized and the product used reasonably ( Sun *et al*., 2015). In the present study, toxicity to gingival fibroblasts was evaluated by the percentage of cell viability ( Poggio *et al*., 2017). Only the chloroform residue of Oxapampa propolis was found to be nontoxic; the results of the MTT assay, with more than 92% cell viability, suggest good biocompatibility. Although few studies have evaluated the cytotoxicity of propolis on this cell line, they show that these are not very toxic ( Pobiega *et al*., 2019; Santos *et al*., 2019).

When evaluating the fractions of the chloroform partition, only the methanolic fraction had an inhibitory effect, with a minimum inhibitory concentration of 0.78 mg/mL on both strains evaluated. Although no studies of Peruvian propolis have been reported, studies of foreign propolis have found similar results ( Nani *et al*., 2018; Zeng & Jiang, 2010), demonstrating that methanolic extraction can produce a higher concentration of compounds with biological activity ( Flemming *et al*., 2016).

Most bacteria reside naturally in accumulations called biofilms, which are substantially more tolerant to antimicrobials than free-floating cells ( Aydin *et al*., 2018). A biofilm is a community of bacteria that have adhered to a surface. Once adhered, the cells begin to secrete extracellular polymeric substances composed of extracellular DNA, polysaccharides, and proteins. These extracellular substances trap nutrients and water within the biofilm, allowing cells to mature in nutrient-rich conditions while protecting them from desiccation, host immune defences, and antimicrobial agents ( Cabral *et al*., 2009). In the present study, when evaluating the effect of the methanolic fraction of propolis on biofilm thickness, both concentrations were found to have a greater effect than chlorhexidine at 120 hours. This result may have occurred because one of the factors contributing to the resistance of biofilm-mediated infections may be a delay in the penetration of loaded antimicrobial agents due to the binding of the robust extracellular polymeric substance matrix, which prevents complete access to the entire biofilm ( Das Neves *et al*., 2016; Li *et al*., 2016). However, unlike chlorhexidine, the propolis fraction is composed of more than one active component, including flavonoids, phenolic acids, terpenes, aromatic acids, and others ( Sha *et al*., 2009). Identifying the exact chemical substances underlying a certain effect, as well as their defined biochemical mechanisms, is difficult ( Hall & Mah, 2017; Khatoon *et al*., 2018).

The PCR results showed that the concentration of 1,563 mg/mL of the methanolic fraction of propolis decreased the copy numbers of the *srtA* gene of *S. gordonii* and the *radD* gene of *F. nucleatum* at 120 hours. Although no previous studies have evaluated the effect of propolis on biofilms of these species, some research suggests that propolis has a dose-dependent effect on the inhibition of genes involved in adherence, showing a potential effect beyond bacterial growth inhibition ( Veloz *et al*., 2019a, 2019b).

The results of component identification by LC/MS of the methanolic fraction of Oxapampa propolis showed the presence of rhamnocitrin, apigenin, kaempferol, isorhamnetin, diosmetin, acacetin, glycitein, and chrysoeriol. The first three compounds have already been described to have antibiofilm effects in previous studies ( Palomino *et al*., 2009). Previous studies have highlighted apigenin as a flavonoid with considerable antibiofilm potency ( Nani *et al.,* 2018; Palomino *et al.,* 2009) due to its ability to increase the permeability of the bacterial membrane, resulting in cellular apoptosis (Zeng *et al*., 2010). Other studies have highlighted the potential of flavonoids to inhibit the formation of the extracellular polymeric matrix, exposing cells that form biofilms and increasing their susceptibility to antimicrobial therapies ( Aydin *et al*., 2018).

The results of this study show a significant antibiofilm effect of Oxapampa propolis without causing damage to oral human cells (gingival fibroblasts), rendering it a promising option for evaluation as a new molecule for the development of new pharmacological agents that may be valuable in the dentistry field.

## Data availability

### Underlying data

Mendeley Data: Databases (antibiofilm assays). https://doi.org/10.17632/3bc8dd6x3t.3 ( Millones-Gómez, 2021).

This project contains the following underlying data:

- Raw microscopy images
- CT data.xlsx
- Raw data.xlsx

Data are available under the terms of the Creative Commons Attribution 4.0 International license (CC-BY 4.0).
