# Supplementary material for: Antibacterial, antibiofilm, and cytotoxic activities and chemical compositions of Peruvian propolis in an in vitro oral biofilm
Source: F1000Res. 2021 Dec 23;10:1093. Originally published 2021 Oct 27. [Version 2] doi: 10.12688/f1000research.73602.2 (PMC8613507; doi:10.12688/f1000research.73602.2)
Supplement: Peer review history

# Peer review: Reviewer response for version 2

**DOI:** 10.5256/f1000research.81399.r117982

**Published:** 2022-02-11

**Authors:** Myriam Angélica de la Garza-Ramos

**Affiliations:** 1 Faculty of Dentistry, Autonomous University of Nuevo Leon, San Nicolás de los Garza, Mexico

The authors of the manuscript demonstrate the ability of Oxapampa propolis to inhibit the growth of two common oral pathogenic bacteria, with minimal effects on gingival fibroblasts at the minimum inhibitory concentration. In addition, the authors demonstrated the potential of Oxapampa propolis to inhibit biofilm formation using in vitro established biofilms of these same bacterial species. These aforementioned effects were found in the methanolic fractions of the chloroform residue of propolis extracts.

 Although further research is warranted on the safety and efficacy of oral therapies, as well as to better understand the bioactive compounds of these extracts (as indicated in the "Discussion" section), this work was logically and rigorously completed and offers valuable insight into a possible use of Oxapampa propolis as a cost-effective and readily available dental therapy presenting sufficient merit for indexing.

Is the work clearly and accurately presented and does it cite the current literature?

Yes

If applicable, is the statistical analysis and its interpretation appropriate?

Yes

Are all the source data underlying the results available to ensure full reproducibility?

Yes

Is the study design appropriate and is the work technically sound?

Yes

Are the conclusions drawn adequately supported by the results?

Yes

Are sufficient details of methods and analysis provided to allow replication by others?

Yes

Reviewer Expertise:

Chemistry, microbiology and molecular biology

I confirm that I have read this submission and believe that I have an appropriate level of expertise to confirm that it is of an acceptable scientific standard.

# Peer review: Reviewer response for version 2

**DOI:** 10.5256/f1000research.81399.r117983

**Published:** 2022-02-11

**Authors:** Luis Maita Castañeda

**Affiliations:** 1 Faculty of Dentistry, National University of San Marcos, Lima, Peru

The manuscript describes the bioactivity of Peruvian propolis extracts against specific bacteria and their ability to inhibit the growth of biofilms of *S. goodonii *and *F. nucleatum*. In particular, the authors selected, among the 13 samples, those with the highest bioactivity and evaluated the crude propolis extract, as well as the fractions, following a specific dissolution procedure.

 The results are promising, in my opinion the work presents significant information on the quality of crude propolis and, in particular, on the composition of each fraction. As mentioned above, the composition of propolis depends to a large extent on the flora surrounding the apiaries, as well as on the climatic and geographical conditions that influence the flora.

Is the work clearly and accurately presented and does it cite the current literature?

Yes

If applicable, is the statistical analysis and its interpretation appropriate?

Yes

Are all the source data underlying the results available to ensure full reproducibility?

Yes

Is the study design appropriate and is the work technically sound?

Yes

Are the conclusions drawn adequately supported by the results?

No

Are sufficient details of methods and analysis provided to allow replication by others?

Yes

Reviewer Expertise:

Studies on natural products

I confirm that I have read this submission and believe that I have an appropriate level of expertise to confirm that it is of an acceptable scientific standard.

## References

[1] : Antibacterial, antibiofilm, and cytotoxic activities and chemical compositions of Peruvian propolis in an in vitro oral biofilm. *F1000Res* .2021;10: 10.12688/f1000research.73602.1 1093 10.12688/f1000research.73602.1 34853678PMC8613507

# Peer review: Reviewer response for version 1

**DOI:** 10.5256/f1000research.77264.r98525

**Published:** 2021-11-24

**Authors:** Myriam Angélica de la Garza-Ramos

**Affiliations:** 1 Faculty of Dentistry, Autonomous University of Nuevo Leon, San Nicolás de los Garza, Mexico

The authors of the manuscript demonstrate the ability of Oxapampa propolis to inhibit the growth of two common oral pathogenic bacteria, with minimal effects on gingival fibroblasts at the minimum inhibitory concentration. In addition, the authors demonstrated the potential of Oxapampa propolis to inhibit biofilm formation using in vitro established biofilms of these same bacterial species. These aforementioned effects were found in the methanolic fractions of the chloroform residue of propolis extracts.

 Although further research is warranted on the safety and efficacy of oral therapies, as well as to better understand the bioactive compounds of these extracts (as indicated in the "Discussion" section), this work was logically and rigorously completed and offers valuable insight into a possible use of Oxapampa propolis as a cost-effective and readily available dental therapy presenting sufficient merit for publication.

Is the work clearly and accurately presented and does it cite the current literature?

Yes

If applicable, is the statistical analysis and its interpretation appropriate?

Yes

Are all the source data underlying the results available to ensure full reproducibility?

Yes

Is the study design appropriate and is the work technically sound?

Yes

Are the conclusions drawn adequately supported by the results?

Yes

Are sufficient details of methods and analysis provided to allow replication by others?

Yes

Reviewer Expertise:

Chemistry, microbiology and molecular biology

I confirm that I have read this submission and believe that I have an appropriate level of expertise to confirm that it is of an acceptable scientific standard.

# Peer review: Reviewer response for version 1

**DOI:** 10.5256/f1000research.77264.r100736

**Published:** 2021-11-22

**Authors:** Luis Maita Castañeda

**Affiliations:** 1 Faculty of Dentistry, National University of San Marcos, Lima, Peru

The manuscript describes the bioactivity of Peruvian propolis extracts against specific bacteria and their ability to inhibit the growth of biofilms of *S. goodonii *and *F. nucleatum*. In particular, the authors selected, among the 13 samples, those with the highest bioactivity and evaluated the crude propolis extract, as well as the fractions, following a specific dissolution procedure.

 The results are promising, in my opinion the work presents significant information on the quality of crude propolis and, in particular, on the composition of each fraction. As mentioned above, the composition of propolis depends to a large extent on the flora surrounding the apiaries, as well as on the climatic and geographical conditions that influence the flora. 

 Translated with www.DeepL.com/Translator (free version).

Is the work clearly and accurately presented and does it cite the current literature?

Yes

If applicable, is the statistical analysis and its interpretation appropriate?

Yes

Are all the source data underlying the results available to ensure full reproducibility?

Yes

Is the study design appropriate and is the work technically sound?

Yes

Are the conclusions drawn adequately supported by the results?

No

Are sufficient details of methods and analysis provided to allow replication by others?

Yes

Reviewer Expertise:

Studies on natural products

I confirm that I have read this submission and believe that I have an appropriate level of expertise to confirm that it is of an acceptable scientific standard.
